# P-1798. Acceptability and Feasibility of Inpatient Group-Level Feedback to Prescribers About Antibiotic Appropriateness for Community-Acquired Pneumonia: A Survey Study

**DOI:** 10.1093/ofid/ofae631.1961

**Published:** 2025-01-29

**Authors:** Kathleen Chiotos, Lauren Dutcher, Ebbing Lautenbach, Melinda M Neuhauser, Keith W Hamilton, Robert Grundmeier, Jeffrey S Gerber, Julia E Szymczak

**Affiliations:** Children's Hospital of Philadelphia, Philadelphia, PA; University of Pennsylvania Perelman School of Medicine, Philadelphia, Pennsylvania; University of Pennsylvania, Philadelphia, Pennsylvania; Division of Healthcare Quality Promotion, Centers for Disease Control and Prevention,, Atlanta, GA; University of Pennsylvania Perelman School of Medicine, Philadelphia, Pennsylvania; Children's Hospital of Philadelphia, Philadelphia, PA; Children's Hospital of Philadelphia, Philadelphia, PA; University of Utah School of Medicine, Salt Lake City, Utah

## Abstract

**Background:**

Feedback on antibiotic prescribing is less commonly used in inpatient settings than outpatient. Our objective was to evaluate the acceptability and feasibility of group-level feedback on antibiotic appropriateness for community-acquired pneumonia (CAP) in two United States hospitals.

**Figure:**
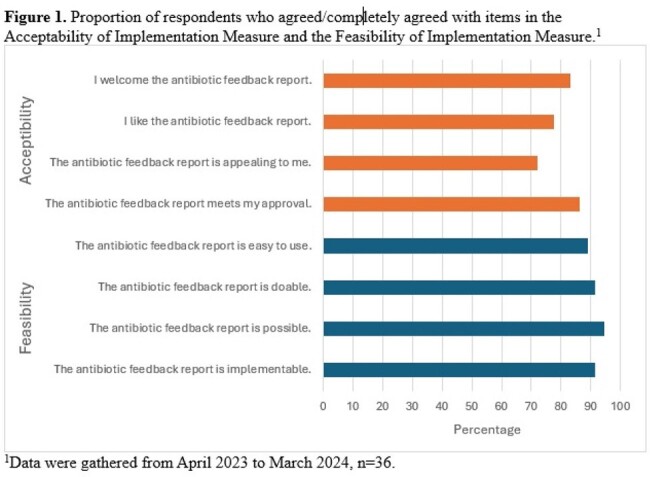

**Methods:**

We conducted a cross-sectional survey of medicine and pediatrics prescribers who received monthly feedback on antibiotic selection and duration for patients with CAP shared in meetings and by email. The survey was administered from April 2023 to March 2024 and included the validated acceptability of intervention measure (AIM) and feasibility of intervention measure (FIM). Each consists of 4 items scored 1-5 and is reported as a mean total score (5-20), with higher scores indicating greater acceptability and feasibility. Data were analyzed using descriptive statistics.

**Results:**

Of 289 eligible prescribers, 36 (12.5%) completed the survey: 28 (77.8%) physicians and 8 (22.2%) advanced practice providers. Engagement with reports was high: 30/35 (85.7%) looked at the reports, and 22/35 (62.8%) attended meetings discussing the report. The majority (25, 69.4%) liked the feedback reports, while 3 (8.3%) disliked the reports, 4 (11.1%) were unsure and 4 (11.1%) did not recall. The majority (n = 26; 76.5%) trusted the reports accurately captured performance. Thirty-four respondents (94.4%) believed the metrics were clinically meaningful, and the majority found them easy to understand: 34 (94.4%) understood choice, while 36 (100%) understood duration. Twenty respondents (55.6%) believed the reports changed how they prescribed, while 9 (25.0%) were unsure. Sixteen respondents (44.4%) believed the reports changed how their colleagues prescribe, while 17 (47.2%) were unsure. Mean totals (SD; range) for acceptability and feasibility were AIM, 17.2 (3.5; 8-20) and FIM, 17.7 (3.1; 8-20).

**Conclusion:**

Although the low response rate may limit generalizability, inpatient clinicians perceived that group-level feedback on antibiotic appropriateness for inpatients with CAP was acceptable and feasible to implement. Clinicians who responded to the survey understood the metrics, found them clinically meaningful, and some felt they influenced prescribing.

**Disclosures:**

**All Authors**: No reported disclosures

